# Soy Food Intake Associated with Obesity and Hypertension in Children and Adolescents in Guangzhou, Southern China

**DOI:** 10.3390/nu14030425

**Published:** 2022-01-18

**Authors:** Xiaotong Wang, Tongtong He, Suhua Xu, Hailin Li, Miao Wu, Zongyu Lin, Fenglian Huang, Yanna Zhu

**Affiliations:** 1 Guangdong Provincial Key Laboratory of Food, Nutrition and Health, Department of Maternal and Child Health, School of Public Health, Sun Yat-sen University, Guangzhou 510080, China; wangxt27@mail2.sysu.edu.cn (X.W.); xush59@mail2.sysu.edu.cn (S.X.); lihlin29@mail2.sysu.edu.cn (H.L.); wumiao6@mail2.sysu.edu.cn (M.W.); linzy35@mail2.sysu.edu.cn (Z.L.); huangflian@mail2.sysu.edu.cn (F.H.); 2 Department of Nutrition, School of Public Health, Sun Yat-sen University, Guangzhou 510080, China; hett5@mail2.sysu.edu.cn

**Keywords:** soy food, obesity, hypertension, children and adolescents

## Abstract

The associations between soy food intake and cardio-metabolic risk factors in children remain unclear due to limited evidence. We aim to explore soy food intake and its association with the risks of obesity and hypertension in Chinese children and adolescents. A total of 10,536 children and adolescents aged 7–18 years (5125 boys and 5411 girls) were enrolled in a cross-sectional study in Guangzhou City, southern China. Data on demographic characteristics and dietary consumption were collected using self-reported questionnaires, and anthropometric characteristics were measured. Obesity, abdominal obesity, and hypertension were defined using Chinese criteria for children and adolescents. A multiple logistic regression model was applied to estimate the association between soy food intake and obesity and hypertension. Roughly 39.5% of the participants consumed soy food more than three times per week. The mean amounts of liquid and solid soy food intake were 0.35 ± 0.54 cups/day and 0.46 ± 0.63 servings/day, respectively. The adjusted odds ratios (OR) of hypertension among those with high liquid soy food intake and a high frequency of all soy food intake (more than three times/week) were 0.79 (95% confidence interval (CI), 0.67–0.94), and 0.83 (95% CI, 0.70–0.97) compared to those with no intake. Additionally, the adjusted OR of obesity among those with high solid soy food intake and a high frequency of all soy food intake were 1.34 (95% CI, 1.09–1.63) and 1.30 (95% CI, 1.07–1.58), respectively. In conclusion, 39.5% of southern Chinese children and adolescents had high soy food intake (more than three times/week), which was significantly associated with a lower prevalence of hypertension and a greater prevalence of obesity.

## 1. Introduction

The prevalence of childhood cardio-metabolic risk factors, specifically obesity and hypertension, are sharply increasing and constitute a major public health threat in China [1,2,3]; these risk factors may persist into adulthood and are the leading causes of cardiovascular disease (CVD) [4,5]. Many studies have reported that a habitual diet is one of the most effective factors in regulating weight and blood pressure (BP) [6,7,8,9]. The consumption of soy food, one of the common habitual diet components, as a valuable source of isoflavones, phytosterols, lecithin, poly-unsaturated fatty acids, dietary fibers, and high-quality protein has attracted significant attention [10] and has demonstrated many health benefits, including a reduction of CVD risk, making soy food worthy of further examination [11,12,13].

Several studies have reported that habitual soy food intake lowers the risk of high BP [14,15,16,17,18,19,20,21,22]. However, some studies observed no significant changes in BP after the consumption of soy food [7,23]. Current epidemiological findings suggest certain effects of soy supplementation on weight control and the prevention of obesity [6,24]. For instance, Ruscica et al. documented significant changes in median percentage of body weight (−1.5%, *p* = 0.005) and body mass index (BMI) (−1.5%, *p* = 0.05) in the soy group [24]. Moreover, another study showed that soy protein is at least as good as other protein sources for weight loss during low-calorie dietary interventions in older adults [11]. Nevertheless, some studies have also shown that the protective effect of legumes on weight remains insufficient [25,26,27]. In addition, a systematic review and meta-analysis suggested that soy had no statistically significant effect on weight in the general population, yet an obesogenic effect of soy was observed in obese subjects (BMI ≥ 30 kg/m^2^) and participants whose daily soy food intake included at least 40 g of soy protein [28].

However, most of these studies have focused on Western populations or older adults; whether the same relationship exists in children remains obscure. Furthermore, there is little information on the association between soy food intake and cardio-metabolic risk factors in children and adolescents; in particular, a better understanding of soy food intake status and its impact on obesity and hypertension, as well as the underlying influencing factors, among children and adolescents is needed. This study, therefore, sought to describe the status of soy food intake and investigate the association of soy food intake with the risk of obesity and hypertension in Chinese children and adolescents aged 7–18 years.

## 2. Materials and Methods

### 2.1. Study Sample

The data were obtained from cross-sectional health and nutrition surveys deployed among children and adolescents in Guangzhou, southern China, in 2018. Fourteen elementary schools and 15 middle schools were randomly selected through multiple cluster sampling, and two classes of each grade from the selected schools were randomly chosen. In total, 13,979 children and adolescents aged 7–18 years old were involved in the present study and asked to undergo anthropometric measurements and questionnaire assessments. Participants were excluded if they did not undergo anthropometric measurements or if their completed questionnaire lacked information on dietary intake or demographic characteristics (*n* = 3443). Finally, the study sample consisted of 10,536 participants (48.6% boys). Informed consent forms were voluntarily signed by all participants and their legal guardians, and the study was approved by the Ethical Committee of School of Public Health, Sun Yat-sen University (Number (2018)005).

### 2.2. Anthropometric Measurements

All participants underwent anthropometric measurements, including height, weight, waist circumference, and blood pressure, which were collected by trained clinicians and nurses according to a uniform standard. Height was measured to the nearest 0.1 cm with the use of a fixed stadiometer (Yilian TZG; Yangzhou, Jiangsu, China) and weight was measured to the nearest 0.1 kg with the use of a lever scale (Hengxing RGT-140; Yixing, Jiangsu, China). Body mass index (BMI) was calculated as the weight divided by the height squared (kg/cm^2^). Waist circumference (WC) was measured to an accuracy of 0.1 cm at 1 cm above umbilicus using a flexible tape in the standing position. Systolic blood pressure (SBP) and diastolic blood pressure (DBP) were measured on the mid-upper right arm using a mercury sphygmomanometer (Yutu XJ1ID; Shanghai, China) after participants sat quietly for at least 5 min. All indices were measured twice and the average numbers were recorded.

### 2.3. Questionnaire Assessment

The self-reported questionnaire in this study was developed based on previously tested and validated questions, including those on demographic information (examination date, sex, birth date, age, and family income) and dietary intake (fruits, vegetables, meat, sugar-sweetened beverages (SSBs), milk, fried food, and soy food intake). The children and adolescents were asked to answer the questionnaire on their own if they were over 9 years old, and children younger than 9 years old were required to bring the questionnaire to their legal guardians. Trained staff and teachers interpreted all of the questions so that the participants understood the questions correctly. The researchers performed quality control when the questionnaires were collected from each class.

To investigate soy food intake and frequency, every participant was required to answer the following questions: “How many times have you consumed soy food in the last week (including soybeans, tofu, soy milk, fermented bean curd, and other products made from soybean)?” and “How many cups of liquid soy food or servings of solid soy food did you consume each time? (Milliliters is equal to one cup, the size of an adult’s palm is equal to one serving)”. The consumption of fruit and vegetables was evaluated using the following two questions: “How many days have you eaten fruits/vegetables in the last week?” and “How many servings of fruits/vegetables did you eat each day? The size of an adult’s fist is equal to one serving”. For meat or meat product consumption, the following two questions were asked: “How many days have you eaten meat or meat products in the last week?” and “How many servings of meat or meat products did you eat last week? The size of an adult’s palm is equal to one serving”. For SSB consumption, the following two questions were asked: “How many days did you drink SSBs in the last week?” and “How many servings of SSB did you drink each day? Two hundred fifty milliliters is equal to one serving”. For this study, SSBs included Coca-Cola (Coca-Cola Company, Atlanta, GA, USA), orange juice, Red Bull (Red Bull GmbH, Fuschl, Austria), and other beverages containing sugar. For milk consumption, the following questions were asked: “How many times did you drink milk in the last week?” and “How many milliliters of milk did you drink each time? Milk includes fresh milk, yogurt, milk powder, and other dairy products”. Finally, data on fried food consumption were collected by asking the following question: “How many times have you eaten fried food in the last week?”. For this study, fried food included fried chicken, fried dough sticks, fried chips, and so on. For demographic information, family income was divided into the following five RMB/month groups: 2000 or below, 2000 to 5000, 5000 to 8000, 8000 or above, and don’t know or no answer.

### 2.4. Definitions

Overweight and obesity were defined by BMIs in at least the 85th percentile and at least the 95th percentile according to the age- and sex-specific cutoff points recommended by the Group of China Obesity Task Force [29]. Abdominal obesity was defined by a WC of at least the 90th age- and sex-specific percentile cutoff point for Chinese children and adolescents [30]. Individuals with SBP and/or DBP values in at least the 90th percentile but less than the 95th percentile for their age and sex were considered to be pre-hypertensive, while those with values in at least the 95th percentile were considered to have hypertension [31].

### 2.5. Statistical Analysis

The data were entered into the EpiData 3.0 software (The EpiData Association, Odense, Denmark). All statistical tests were performed using the Statistical Package for the Social Sciences version 21.0 software (IBM Corporation, Armonk, NY, USA). The participants were classified into three groups according to tertile cutoff points of soy food intake, as follows: (1) liquid soy food intake (none = 0 cups/day, medium = 0–0.4 cups/day, high > 0.4 cups/day); (2) solid soy food intake (none = 0–0.1429 servings/day, medium = 0.1429–0.4286 servings/day, high > 0.4286 servings/day); (3) total soy food intake frequency (none = 0–1 times/week, medium = 1–3 times/week, high > 3 times/week). The average soy food intake by age is shown using mean ± and standard deviation values, and the characteristics of the participants stratified by soy food intake are presented as mean ± standard error values for quantitative variables or numbers and percentages for the categorical variables. The *t* test was used to compare differences in soy food intake between boys and girls. When assessing the differences of the characteristics among the three groups of soy food intake, a one-way analysis of variance (ANOVA) was applied for quantitative variables, and a non-parametric test was applied if the normality test and homogeneity test of variances were unsatisfied; meanwhile, a χchi-squared test was applied for the categorical variables. Bonferroni’s test was used to analyze pairwise comparisons. The association of soy food intake with obesity and hypertension was evaluated by using multivariate logistic regression in four models (model 1, unadjusted; model 2, adjusted for age and sex; model 3, model 2 plus adjustments for family income and overweight, obesity, and abdominal obesity, and family income and BMI for blood pressure; model 4, model 3 plus adjustments for dietary intakes (intake frequency of fried food and SSBs)). The results were reported with an odds ratio (OR) and corresponding 95% confidence interval (95% CI) values, and *p* values of less than 0.05 were considered to be statistically significant.

## 3. Results

### 3.1. Soy Food Intake in Chinese Children and Adolescents According to Age

In this cross-sectional study, a total of 10,536 Chinese children and adolescents (5125 boys and 5411 girls) aged 7–18 years old were included. Table 1 shows the status of soy food intake according to age and sex. The mean liquid and solid soy food intakes of boys and girls were 0.35 ± 0.54 cups/day and 0.46 ± 0.63 servings/day, respectively. The overall frequency of all soy products was 2.41 ± 2.00 times/week among participants. The servings and frequency of soy food intake were higher in boys compared to those in girls (all *p* < 0.05). The trend showed that boys and girls tended to have more soy food intake as they grew up (both *p* < 0.001), especially in the middle of adolescence.

**Figure 1:**
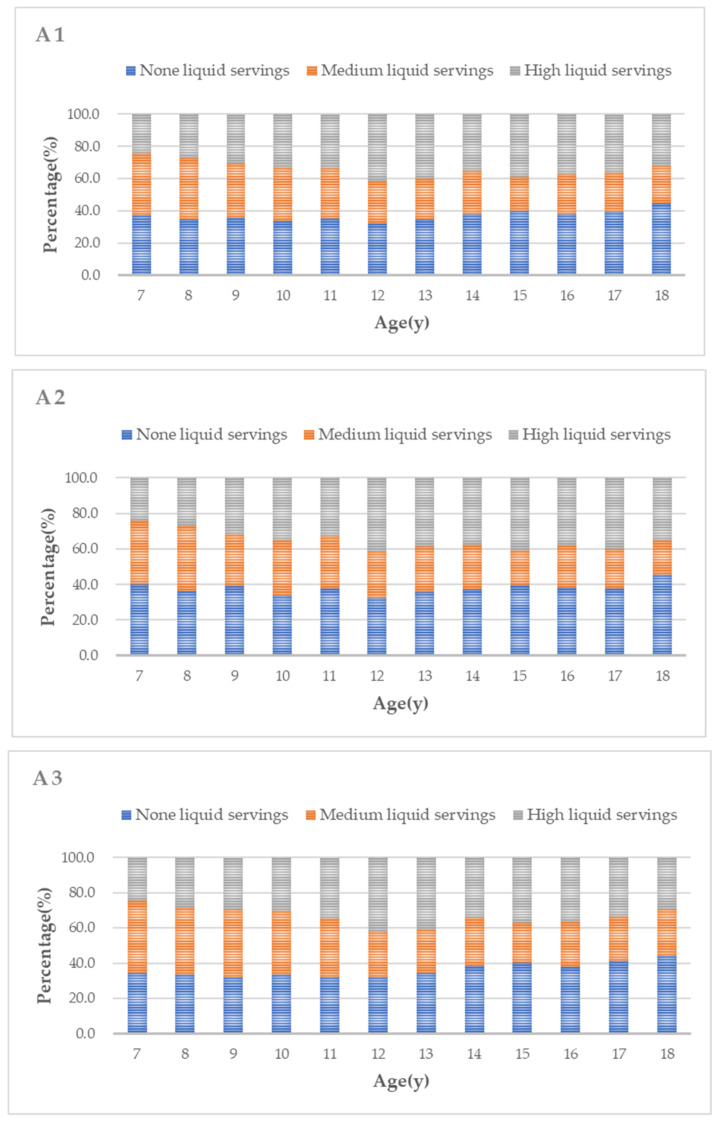

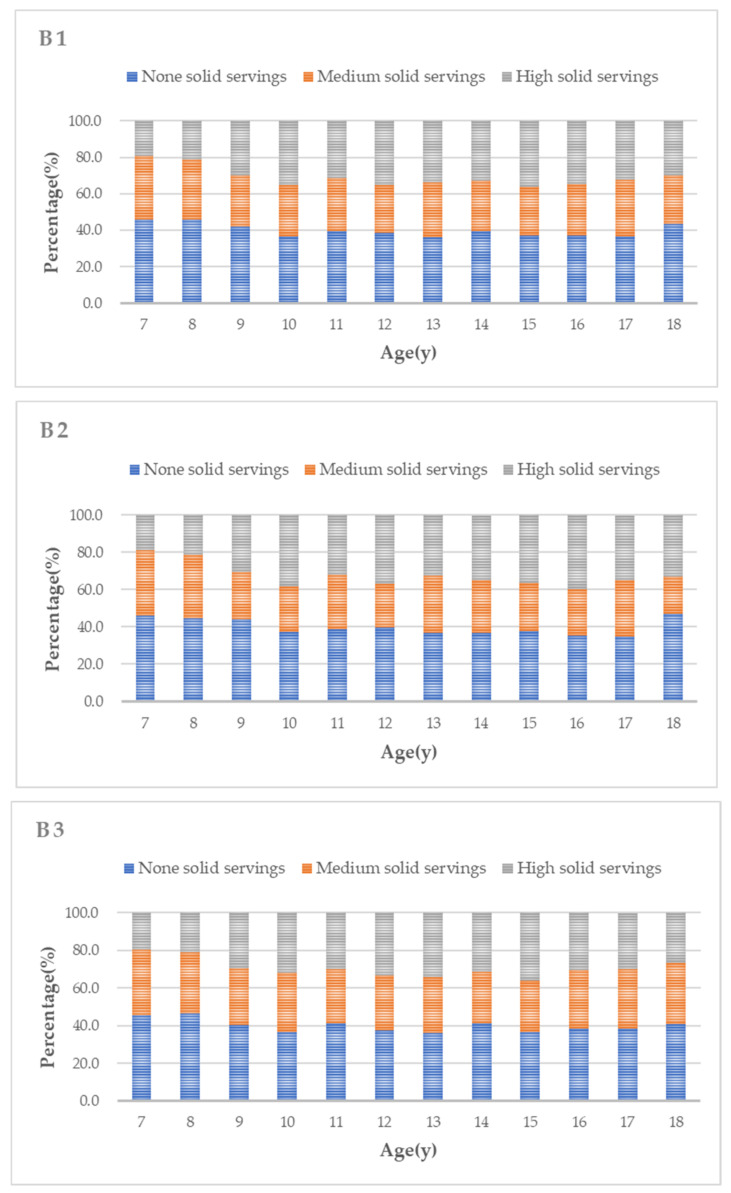

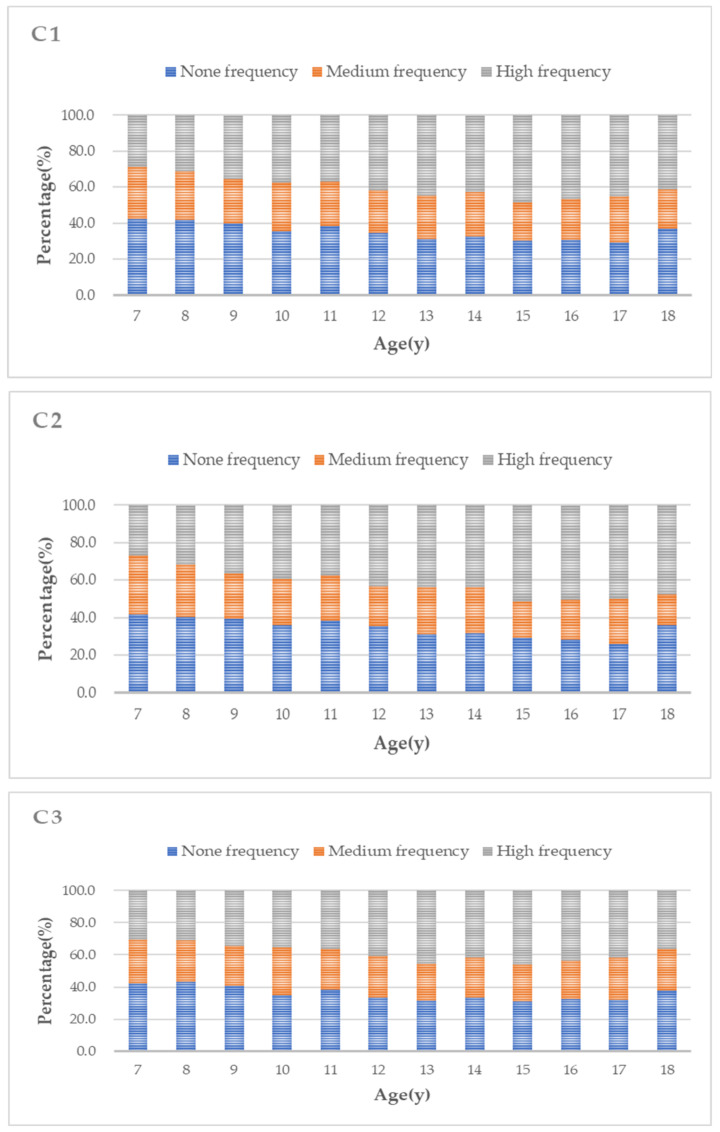
Soy food intake in Chinese children and adolescents according to age 1 for total, 2 for males and 3 for females; (**A1**–**A3**) present data on liquid soy food; (**B1**–**B3**) present data on solid soy food; (**C1**–**C3**) present data on the intake frequency of all soy food. (**A1**,**B1**,**C1**) show data for the total population; (**A2**,**B2**,**C2**) show data for the boys; (**A3**,**B3**,**C3**) show data for the girls.

As shown in Figure 1A1–C3, about 39.5% of participants consumed soy food more than three times per week. Boys tended to increase their liquid soy food intake as they grew up (*p* < 0.01). In general, the proportion of those with higher soy food intake frequency tended to increase with age (all *p* < 0.001), while there was no significant change in solid soy food intake with age.

### 3.2. Characteristics of Participants by Soy Food Intake Levels

The characteristics of the participants according to the tertiles of the liquid and solid servings and the frequency of soy food intake are described in Table 2, including sex, anthropometry, family income, and dietary intake. A high soy food intake and high soy food frequency were associated with higher weight, BMI, QC, and SBP (all *p* < 0.001). Meanwhile, the high servings and high frequency groups showed greater dietary intake, including fruits, vegetables, meat, milk, SSBs, and fried food. No significant differences were found in terms of family income and DBP level among the groups stratified by soy food intake or frequency.

### 3.3. The Distribution of Obesity and Blood Pressure Stratified by Soy Food Intake

The distributions of obesity and blood pressure according to soy food intake are shown in Table 3. Compared with no servings, a high liquid soy food intake was associated with a lower prevalence of normal BMI and a higher prevalence of hypertension (both *p* < 0.05), and children who consumed a high number of servings of solid soy food were more likely to be obese (*p* = 0.032). In addition, the prevalence of pre-hypertension was higher in the high-frequency group (*p* < 0.05). However, soy food intake showed no association with abdominal obesity.

### 3.4. ORs (95% CIs) for Obesity and Hypertension across Soy Food Intake

The association between the risks of obesity, abdominal obesity, and hypertension in children and adolescents across the soy food intake categories is shown in Table 4. The crude OR of hypertension to high servings of liquid soy food intake compared to no liquid servings was 0.840 (95% CI, 0.717–0.984). After adjustment for age, sex, BMI, family income, and dietary intake, compared with no consumption, the OR for hypertension in the high servings of liquid soy food intake and high frequency of the all soy food intake group were 0.790 (95% CI, 0.666–0.937) and 0.828 (95% CI, 0.704–0.974), respectively. The crude OR of obesity to high solid servings compared to no solid servings was 1.287 (95% CI, 1.062–1.559), and the adjusted OR was 1.335 (95% CI, 1.091–1.633). The adjusted OR for obesity in the high frequency of the all soy food intake group was 1.302 (95% CI, 1.070–1.583). However, no significant correlation was found between soy food intake and abdominal obesity.

## 4. Discussion

With the sharply increasing prevalence of childhood obesity and hypertension in recent years [32], several studies have explored the effect of habitual diet in regulating weight and BP. However, the association between soy food intake and cardio-metabolic risk factors in children and adolescents remains unclear due to limited evidence. Using a cross-sectional survey of 10,536 children and adolescents aged 7–18 years old, we analyzed the daily soy food intake and determined that high soy food intake (>3 times/week) was correlated with a higher prevalence of obesity and lower prevalence of hypertension independent of family income and consumption of meat and fried foods. However, no association between soy food and abdominal obesity was observed.

In this study, the mean intakes of liquid and solid soy food were 0.35 ± 0.54 cups/day and 0.46 ± 0.63 servings/day, respectively, with 39.5% of participants consuming soy food more than 3 times per week. The overall frequency of all soy products was 2.41 times per week among participants. Hsiao reported a mean daily soy food intake of 150.1 g among children aged 8–9 years old in Taiwan [33], which is similar to the intake estimated by our study. Daily soy food intake was less than 61 g for 7–14 years old participants in Japan [34], but higher than 40 g of soymilk in 4–14-year-olds in Hong Kong [35]. Nevertheless, Leung et al. reported that, in Hong Kong, young boys had approximately 10 soy-containing meals per week [35], which was much higher than the consumption in our study. In addition, a survey of almost 200 12-year-olds in Beijing found that 41% normally included soymilk or tofu at breakfast [36]. These findings revealed that soy food intake by children was quite uneven across Asian countries [37], even in China. This can be partly explained by regional differences in dietary patterns and a lack of information about soy consumption by children relative to adults.

The present study has shown that habitual soy food intake lowers the risk of high BP. A meta-analysis of 12 trials indicates that the ingestion of at least 25 g of soy protein per day has BP-lowering effects [22], which is consistent with our findings. Additionally, a prospective cohort study in Japan demonstrated that the intake of fermented soy products showed a protecting effect of BP in healthy populations [21]. Soy and some of its constituents mitigate hypertension through the effects of vasodilation [10], which may be caused by isoflavones [38]. However, clinical evidence supporting the underlying mechanisms remains controversial. There are also some studies showing that no significant changes in BP could be observed after the consumption of soy food [7,23].

A growing body of literature suggests soy supplementation affects weight control and obesity prevention [6,24,39,40,41]. For instance, in a 12-week randomized controlled trial, Beavers et al. proposed that soy protein is at least as good as other protein sources for weight loss during low-calorie dietary interventions in older adults [11]. Another study recorded significant changes in the median percentage of body weight (−1.5%, *p* = 0.005) and BMI (−1.5%, *p* = 0.05) in the soy group [24]. There are also studies reporting that the protective effect of legumes, including soy food, beans, and soy dietary supplements, on weight is still insufficient [25,26,27]. Moreover, some studies present conflicting results. For example, a systematic review and meta-analysis suggested that soy showed no statistically significant effect on weight in the general population, but an obesogenic effect of soy was observed in obese subjects (BMI ≥ 30 kg/m^2^) and participants whose daily soy food intake included at least 40 g of soy protein [28], which may be explained by the fact that obese people have less control over their diet and tend to consume more amounts of various foods in daily life compared to people in the normal weight range. Our findings, in part, were consistent with this previous study in that greater soy food intake (>3 times/week) was associated with an increased risk of obesity. In summary, these controversial findings might be due to the weight-loss property of isoflavones [42] and the weight-gain effect of a high amount of soy consumption. Briefly, soy and isoflavones have different impacts on weight status [28].

However, no significant associations were observed between soy food intake and abdominal obesity in this study or in previous studies [28] conducted in children.

Our study has several strengths. First, the current study was based on a large sample of children and adolescents across a wide age range living in Guangzhou City, which is the third-largest city in China and the largest city in southern China. It has a large immigrant population; this could assure the reliability of the data and widen the application of our results. Second, we analyzed the association between the consumption of soy food and cardio-metabolic risk factors in a pediatric population stratified by age and sex. Still, several limitations of the study should not be overlooked. First, as our study applied a cross-sectional analysis, the causality of the relationships observed could hardly be determined with certainty. Second, self-reported questionnaires were used to collect the data on dietary information of the previous week; hence, the data might be affected by recall bias. Finally, although we controlled for several dietary and other potential confounding factors, residual confounding by unknown or unmeasured factors might have also been present.

## 5. Conclusions

In conclusion, 39.5% of 10,536 children and adolescents aged 7–18 years living in Guangzhou City, southern China, had high soy food intake (>3 times/week), and this was significantly associated with a lower prevalence of hypertension and higher prevalence of obesity. Our results may provide a new diet intervention method to reduce the risk of hypertension in childhood and associated cardiovascular diseases in adulthood. However, large prospective studies need to be carried out to confirm the present findings, which can be useful in clarifying the mechanisms and mitigating the current epidemics of childhood obesity and hypertension.

## Figures and Tables

**Table 1:** Soy food intake in Chinese children and adolescents according to age (*n* = 10,536).

Age (y)	Liquid Soy Food (Cups/Day) Mean ± SD			Solid Soy Food (Servings/Day) Mean ± SD			All Soy Food (Times/Week) Mean ± SD		
	Total *N* = 10,536	Boys *n* = 5125	Girls *n* = 5411	Total *N* = 10,536	Boys *n* = 5125	Girls *n* = 5411	Total *N* = 10,536	Boys *n* = 5125	Girls *n* = 5411
7	0.26 ± 0.44	0.26 ± 0.44	0.27 ± 0.46	0.33 ± 0.47	0.33 ± 0.44	0.34 ± 0.51	2.01 ± 1.69	1.95 ± 1.60	2.08 ± 1.79
8	0.27 ± 0.38	0.25 ± 0.31	0.29 ± 0.44	0.35 ± 0.51	0.35 ± 0.46	0.36 ± 0.55	2.05 ± 1.65	2.06 ± 1.69	2.04 ± 1.60
9	0.31 ± 0.47	0.34 ± 0.57	0.28 ± 0.34 *	0.47 ± 0.70	0.48 ± 0.74	0.47 ± 0.66	2.23 ± 1.87	2.26 ± 1.97	2.19 ± 1.75
10	0.35 ± 0.51	0.38 ± 0.55	0.32 ± 0.46 *	0.52 ± 0.66	0.55 ± 0.69	0.49 ± 0.63	2.39 ± 2.02	2.49 ± 2.23	2.27 ± 1.76
11	0.38 ± 0.61	0.38 ± 0.64	0.37 ± 0.59	0.48 ± 0.69	0.52 ± 0.77	0.44 ± 0.58	2.34 ± 2.09	2.38 ± 2.26	2.29 ± 1.86
12	0.43 ± 0.56	0.48 ± 0.63	0.39 ± 0.49 *	0.49 ± 0.61	0.51 ± 0.69	0.46 ± 0.52	2.50 ± 1.96	2.61 ± 1.14	2.40 ± 1.77
13	0.44 ± 0.71	0.46 ± 0.82	0.42 ± 0.61	0.49 ± 0.65	0.53 ± 0.77	0.47 ± 0.54	2.59 ± 2.07	2.62 ± 2.29	2.57 ± 1.90
14	0.33 ± 0.43	0.36 ± 0.47	0.30 ± 0.40	0.46 ± 0.60	0.52 ± 0.66	0.42 ± 0.56 *	2.51 ± 2.06	2.63 ± 2.11	2.41 ± 2.02
15	0.38 ± 0.57	0.43 ± 0.62	0.35 ± 0.52 *	0.50 ± 0.65	0.53 ± 0.72	0.47 ± 0.58	2.71 ± 2.14	2.92 ± 2.28	2.53 ± 2.01 *
16	0.40 ± 0.61	0.43 ± 0.67	0.37 ± 0.56	0.50 ± 0.64	0.55 ± 0.68	0.47 ± 0.61	2.72 ± 2.18	2.98 ± 2.45	2.51 ± 1.91 *
17	0.37 ± 0.58	0.42 ± 0.64	0.34 ± 0.52	0.49 ± 0.66	0.54 ± 0.74	0.46 ± 0.59	2.65 ± 2.04	2.82 ± 2.08	2.52 ± 2.00 *
18	0.33 ± 0.51	0.38 ± 0.56	0.28 ± 0.44	0.44 ± 0.66	0.43 ± 0.52	0.46 ± 0.76	2.52 ± 2.20	2.76 ± 2.45	2.31 ± 1.94
Total	0.35 ± 0.54	0.37 ± 0.58	0.33 ± 0.50 *	0.46 ± 0.63	0.48 ± 0.67	0.44 ± 0.59 *	2.41 ± 2.00	2.48 ± 2.13	2.32 ± 1.86 *

Abbreviation: SD, standard deviation. Liquid soy food (cups/day) = liquid soy food (cups/time) * intake of liquid soy food (times/week)/7. Solid soy food (servings/day) = solid soy food (servings/time) * intake of solid soy food (times/week)/7. * *p* < 0.05, *t* test compared the differences between boys and girls in soy food intake.

**Table 2:** Characteristics of participants stratified by soy food intake level.

Variables	Liquid Soy Food (Cups/Day)				Solid Soy Food (Servings/Day)				All Soy Food (Times/Week)			
	None ^1,^^†^	Medium ^1,^^†^	High ^1,^^†^	*p* Value	None ^2,^^†^	Medium ^2,^^†^	High ^2,^^†^	*p* Value	None ^3,^^†^	Medium ^3,^^†^	High ^3,^^†^	*p* Value
Sex, %				0.010 ^b^				0.011 ^b^				0.127
Male	37.3	28.5 *	34.2		40.0	28.3	31.7		35.1	24.4	40.4	
Female	35.6	31.2 *	32.2		40.1	30.5	29.4		36.0	25.5	38.5	
Family income, RMB/month, %				0.320				0.009 ^b^				0.038 ^b^
2000 or below	35.5	29.8	34.6	0.880	44.9	25.1 *	30.1	0.052	39.7	21.1 *	39.2	0.076
2000–5000	34.4	30.8	34.8 *	0.061	38.7	29.6	31.7	0.219	34.3	26.1	39.6	0.233
5000–8000	36.5	30.9	32.6	0.416	38.9	31.2	29.9	0.115	36.6	26.2	37.2 *	0.046 ^b^
≥8000	37.2	29.6	33.2	0.525	39.8	30.3	29.9	0.376	34.9	25.0	40.0	0.640
Don’t know or no answer	37.7	28.4	33.6	0.214	42.4	26.8 *	30.9	0.009 ^b^	35.9	23.2	40.9	0.098
Anthropometry, mean ± SE												
Weight (kg)	42.62 ± 0.23	39.53 ± 0.26 *	43.77 ± 0.25 *	<0.001 ^a^	41.21 ± 0.23	41.14 ± 0.26	44.08 ± 0.26 *	<0.001 ^a^	40.51 ± 0.24	40.83 ± 0.28	44.30 ± 0.23 *	<0.001 ^a^
BMI (kg/m^2^)	18.30 ± 0.06	17.78 ± 0.06 *	18.52 ± 0.06 *	<0.001 ^a^	18.03 ± 0.05	18.07 ± 0.06	18.61 ± 0.07 *	<0.001 ^a^	17.94 ± 0.06	17.99 ± 0.07	18.63 ± 0.06 *	<0.001 ^a^
WC (cm)	64.11 ± 0.16	62.52 ± 0.18 *	64.57 ± 0.16	<0.001 ^a^	63.30 ± 0.15	63.30 ± 0.17	64.91 ± 0.18 *	<0.001 ^a^	63.04 ± 0.16	63.22 ± 0.19	64.84 ± 0.15 *	<0.001 ^a^
SBP (mmHg)	105.97 ± 0.19	104.91 ± 0.21 *	106.24 ± 0.19	<0.001 ^a^	105.35 ± 0.18	105.55 ± 0.21	106.44 ± 0.21 *	<0.001 ^a^	105.05 ± 0.19	105.63 ± 0.23	106.43 ± 0.18 *	<0.001 ^a^
DBP (mmHg)	65.81 ± 0.13	65.65 ± 0.15	65.89 ± 0.13	0.471	65.71 ± 0.12	65.62 ± 0.15	66.05 ± 0.14	0.082	65.59 ± 0.13	65.93 ± 0.16	65.87 ± 0.13	0.171
Dietary conditions, mean ± SE												
Fruit, servings/day	1.06 ± 0.02	1.09 ± 0.02 *	1.25 ± 0.02 *	<0.001 ^c^	1.05 ± 0.02	1.08 ± 0.02 *	1.28 ± 0.02 *	<0.001 ^c^	1.07 ± 0.02	1.09 ± 0.02 *	1.22 ± 0.02 *	<0.001 ^c^
Vegetables, servings/day	1.84 ± 0.02	1.82 ± 0.03	2.01 ± 0.03 *	<0.001 ^c^	1.76 ± 0.02	1.79 ± 0.02 *	2.17 ± 0.03 *	<0.001 ^c^	1.79 ± 0.03	1.90 ± 0.03 *	1.98 ± 0.02 *	<0.001 ^c^
Meat, servings/day	1.71 ± 0.02	1.59 ± 0.02 *	1.79 ± 0.03 *	<0.001 ^c^	1.64 ± 0.02	1.53 ± 0.02	1.94 ± 0.03 *	<0.001 ^c^	1.67 ± 0.02	1.65 ± 0.03	1.76 ± 0.02 *	<0.001 ^c^
Milk, servings/day	0.61 ± 0.01	0.59 ± 0.01	0.72 ± 0.01 *	<0.001 ^c^	0.60 ± 0.01	0.63 ± 0.01 *	0.71 ± 0.10 *	<0.001 ^c^	0.58 ± 0.01	0.61 ± 0.01 *	0.71 ± 0.01 *	<0.001 ^c^
SSBs, servings/day	0.21 ± 0.01	0.25 ± 0.01 *	0.32 ± 0.01 *	<0.001 ^c^	0.24 ± 0.01	0.24 ± 0.01 *	0.31 ± 0.01 *	<0.001 ^c^	0.24 ± 0.01	0.24 ± 0.01 *	0.29 ± 0.01 *	<0.001 ^c^
Fried food times/week	0.62 ± 0.02	0.82 ± 0.02 *	1.00 ± 0.02 *	<0.001 ^c^	0.67 ± 0.02	0.81 ± 0.02 *	0.99 ± 0.03 *	<0.001 ^c^	0.68 ± 0.02	0.77 ± 0.02 *	0.95 ± 0.02 *	<0.001 ^c^

Abbreviations: BMI, body mass index; DBP, diastolic blood pressure; SBP, systolic blood pressure; SE, standard error; SSB, sugar-sweetened beverage; WC, waist circumference; ANOVA, analysis of variance. ^†^ None ^1^, 0 cups/day; medium ^1^, 0–0.4 cups/day; high ^1^, >0.4 cups/day. None ^2^, 0–0.1429 servings/day; medium ^2^, 0.1429–0.4286 servings/day; high ^2^, >0.4286 servings/day. None ^3^, 0–1 times/week; medium ^3^, 1–3 times/week; high ^3^, >3 times/week. * *p* < 0.05, compared with no intake (χ2 test or one-way ANOVA test to discriminate pairwise comparisons). ^a^ *p* < 0.05, boys vs. girls, assessed by one-way ANOVA. ^b^ *p* < 0.05, boys vs. girls, assessed by the χ2 test for categorical variables. ^c^ *p* < 0.05, boys vs. girls, assessed by the Kruskal–Wallis H test.

**Table 3:** The distribution of obesity and blood pressure stratified by soy food intake (%).

		Liquid Soy Food (Cups/Day)				Solid Soy Food (Servings/Day)				All Soy Food (Times/Week)			
	Total	None ^1,^^†^	Medium ^1,^^†^	High ^1,^^†^	*p* Value	None ^2,^^†^	Medium ^2,^^†^	High ^2,^^†^	*p* Value	None ^3,^^†^	Medium ^3,^^†^	High ^3,^^†^	*p* Value
BMI					0.004 ^a^				0.059				0.105
Normal, %	70.6	72.0	68.8 *	70.7	0.013 ^a^	71.2	70.5	69.8	0.381	70.5	70.8	70.4	0.935
Overweight, %	9.5	9.5	9.0	9.9	0.448	9.4	8.8	10.3	0.139	9.6	8.6	10.0	0.138
Obesity, %	6.2	5.8	6.5	6.3	0.414	5.5	6.5	6.9 *	0.032 ^a^	5.7	6.2	6.7	0.175
Abdominal obesity					0.639				0.143				0.074
No, %	86.1	86.5	86.0	85.7		86.4	86.6	85.1		86.2	87.2	85.3	
Yes, %	13.9	13.5	14.0	14.3		13.6	13.4	14.9		13.8	12.8	14.7	
Hypertension HTN					0.038 ^a^				0.263				0.116
Normal, %	75.6	75.3	75.3	76.3	0.569	75.9	76.1	74.9	0.528	76.0	75.2	75.6	0.750
Pre-hypertension, %	14.5	14.5	13.9	15.1	0.395	14.0	14.0	15.6	0.095	13.7	14.4	15.3 *	0.142
Hypertension, %	9.9	10.2	10.8	8.7 *	0.010 ^a^	10.1	9.9	9.4	0.600	10.3	10.5	9.1	0.114

Abbreviations: BMI, body mass index; DBP, diastolic blood pressure; SBP, systolic blood pressure. ^†^ None ^1^, 0 cups/day; medium ^1^, 0–0.4 cups/day; high ^1^, >0.4 cups/day. None ^2^, 0–0.1429 servings/day; medium ^2^, 0.1429–0.4286 servings/day; high ^2^, >0.4286 servings/day. None ^3^, 0–1 times/week; medium ^3^, 1–3 times/week; high ^3^, >3 times/week. * *p* < 0.05, compared with no intake (χ2 test to discriminate pairwise comparisons). ^a^ *p* < 0.05, boys vs. girls, assessed by the χ2 test for categorical variables.

**Table 4:** Odds ratios (95% confidence intervals) for obesity and hypertension across soy food intake groups.

	Overweight OR (95% CI)	Obesity OR (95% CI)	Abdominal Obesity OR (95% CI)	Pre-Hypertension OR (95% CI)	Hypertension OR (95% CI)
Liquid soy food (cups/day) ^a^					
Model 1					
No servings	1	1	1	1	1
Medium servings	0.987 (0.837–1.164)	1.179 (0.968–1.436)	1.044 (0.911–1.197)	0.960 (0.837–1.101)	1.057 (0.906–1.235)
High servings	1.057 (0.904–1.235)	1.112 (0.917–1.348)	1.064 (0.933–1.214)	1.028 (0.903–1.171)	0.840 (0.717–0.984) *
Model 2					
No servings	1	1	1	1	1
Medium servings	0.994 (0.841–1.175)	1.141 (0.934–1.394)	1.026 (0.894–1.177)	1.098 (0.954–1.263)	1.076 (0.920–1.257)
High servings	1.071 (0.915–1.253)	1.154 (0.949–1.402)	1.071 (0.939–1.223)	1.000 (0.876–1.142)	0.839 (0.716–0.983) *
Model 3					
No servings	1	1	1	1	1
Medium servings	1.009 (0.852–1.239)	1.163 (0.950–1.425)	1.027 (0.893–1.180)	1.123 (0.973–1.297)	1.116 (0.949–1.312)
High servings	1.072 (0.913–1.259)	1.175 (0.964–1.431)	1.079 (0.944–1.234)	0.985 (0.860–1.129)	0.804 (0.681–0.948) *
Model 4					
No servings	1	1	1	1	1
Medium servings	1.067 (0.896–1.272)	1.131 (0.918–1.394)	1.032 (0.893–1.192)	1.091 (0.941–1.265)	1.112 (0.941–1.313)
High servings	1.123 (0.951–1.327)	1.194 (0.975–1.461)	1.124 (0.979–1.291)	0.958 (0.832–1.102)	0.790 (0.666–0.937) *
Solid soy food (servings/day) ^b^					
Model 1					
No servings	1	1	1	1	1
Medium servings	0.944 (0.802–1.112)	1.195 (0.981–1.455)	0.984 (0.859–1.127)	0.997 (0.871–1.141)	0.975 (0.835–1.140)
High servings	1.113 (0.952–1.301)	1.287 (1.062–1.559) *	1.118 (0.980–1.274)	1.129 (0.991–1.286)	0.942 (0.806–1.102)
Model 2					
No servings	1	1	1	1	1
Medium servings	0.961 (0.815–1.134)	1.220 (1.000–1.488)	0.986 (0.861–1.130)	1.005 (0.876–1.154)	0.981 (0.839–1.146)
High servings	1.123 (0.959–1.315)	1.365 (1.123–1.659) *	1.137 (0.997–1.297)	1.027 (0.898–1.173)	0.934 (0.798–1.093)
Model 3					
No servings	1	1	1	1	1
Medium servings	0.974 (0.823–1.152)	1.212 (0.992–1.481)	0.974 (0.848–1.118)	1.000 (0.868–1.151)	0.980 (0.834–1.152)
High servings	1.132 (0.964–1.330)	1.330 (1.092–1.620) *	1.123 (0.983–1.283)	0.997 (0.87–1.143)	0.866 (0.735–1.020)
Model 4					
No servings	1	1	1	1	1
Medium servings	1.008 (0.848–1.199)	1.189 (0.967–1.461)	0.968 (0.840–1.116)	0.974 (0.842–1.127)	0.985 (0.834–1.163)
High servings	1.158 (0.981–1.367)	1.335 (1.091–1.633) *	1.147 (1.000–1.315)	1.006 (0.874–1.158)	0.867 (0.732–1.027)
All soy food (times/week) ^c^					
Model 1					
No frequency	1	1	1	1	1
Medium frequency	0.887 (0.743–1.059)	1.086 (0.878–1.343)	0.912 (0.787–1.056)	1.060 (0.917–1.225)	1.027 (0.871–1.211)
High frequency	1.042 (0897–1.212)	1.180 (0.980–1.422)	1.076 (0.948–1.221)	1.120 (0.986–1.271)	0.891 (0.767–1.036)
Model 2					
No frequency	1	1	1	1	1
Medium frequency	0.894 (0.748–1.069)	1.104 (0.891–1.368)	0.916 (0.790–1.061)	1.043 (0.899–1.209)	1.027 (0.871–1.212)
High frequency	1.061 (0.910–1.236)	1.281 (1.060–1.548) *	1.103 (0.971–1.253)	0.972 (0.853–1.108)	0.881 (0.757–1.025)
Model 3					
No frequency	1	1	1	1	1
Medium frequency	0.884 (0.737–1.061)	1.119 (0.902–1.389)	0.898 (0.773–1.043)	1.059 (0.911–1.232)	1.034 (0.871–1.227)
High frequency	1.062 (0.909–1.241)	1.256 (1.036–1.521) *	1.089 (0.957–1.239)	0.938 (0.821–1.073)	0.827 (0.706–0.968) *
Model 4					
No frequency	1	1	1	1	1
Medium frequency	0.896 (0.743–1.081)	1.089 (0.871–1.361)	0.887 (0.760–1.036)	1.072 (0.918–1.252)	1.051 (0.881–1.254)
High frequency	1.087 (0.926–1.277)	1.302 (1.070–1.583) *	1.119 (0.981–1.278)	0.937 (0.816–1.075)	0.828 (0.704–0.974) *

* *p* < 0.05. ^a^ None ^1^, 0 cups/day; medium ^1^, 0–0.4 cups/day; high ^1^, > 0.4 cups/day; ^b^ None ^2^, 0–0.1429 servings/day; medium ^2^, 0.1429–0.4286 servings/day; high ^2^, > 0.4286 servings/day. ^c^ None ^3^, 0–1 times/week; medium ^3^, 1–3 times/week; high ^3^, >3 times/week. Model 1 is without adjustment; model 2 is adjusted for age and sex; model 3 is additionally adjusted for family income for overweight, obesity, and abdominal obesity and family income and BMI for blood pressure; model 4 is additionally adjusted for dietary intakes (intake frequency of fried food and SSB).

## Data Availability

All data generated or analyzed during this study are included in this article. Further inquiries can be directed to the corresponding author.
